# Formal Comment to Gong et al.: Ecosystem Scale Acoustic Sensing Reveals Humpback Whale Behavior Synchronous with Herring Spawning Processes and Re-Evaluation Finds No Effect of Sonar on Humpback Song Occurrence in the Gulf of Maine in Fall 2006

**DOI:** 10.1371/journal.pone.0109225

**Published:** 2014-10-07

**Authors:** Denise Risch, Peter J. Corkeron, William T. Ellison, Sofie M. Van Parijs

**Affiliations:** 1 Integrated Statistics, Woods Hole, Massachusetts, United States of America; 2 Northeast Fisheries Science Center, Woods Hole, Massachusetts, United States of America; 3 Marine Acoustics, Inc., Middletown, Rhode Island, United States of America; Pacific Northwest National Laboratory, United States of America

## Introduction

In their paper: “Ecosystem scale acoustic sensing reveals humpback whale behavior synchronous with herring spawning processes and re-evaluation finds no effect of sonar on humpback song occurrence in the Gulf of Maine in Fall 2006” Gong et al. [1] used acoustic monitoring to detect synchronous herring spawning and humpback whale presence in the Gulf of Maine, presenting novel and interesting data on predator-prey relationships on a major humpback whale feeding ground in the western North Atlantic. In addition, their finding that singing humpback whales recorded on Georges Bank and in the vicinity to their active Ocean Acoustic Waveguide Remote Sensing (OAWRS) source array did not seem to respond acoustically to OAWRS transmissions during a Fall 2006 experiment is also new. Gong et al. [1] argue that it is different from our observations in the Stellwagen Bank National Marine Sanctuary (SBNMS), 200 km distant from the OAWRS source array during the same experiment [2].

However, in contrast to the conclusions of Gong et al. [1], we argue that their results do not contradict, nor necessarily conflict with, our findings. Variable behavioral responses to noise have been shown in a range of marine mammals. For example blue whales have been shown both to increase and decrease calling rates in response to different noise sources [3], [4]. Responses to noise are complex in nature and may depend on factors such as behavioral context, prey availability, distance from source, received level (RL), signal structure and novelty, as well as individual differences [5], [6]. We argue that the finding that humpback whales reacted differently towards the OAWRS signal depending on range to the source, RL above background noise and (likely) differences in behavioral state is an interesting result that should be highlighted rather than discounted.

Here we will (1) outline some of the problems in the Gong et al. [1] analysis of humpback whale vocal behavior on Georges Bank, (2) respond to their critique of our findings reported in [2], and (3) discuss Gong et al.'s [1] presentation of the effects of the OAWRS signal source, a low-frequency sound source with source levels (SL) which may be in excess of 210 dB re 1 µPa @ 1 m [7].

## Analysis of Humpback Whale Vocalizations

### 1. Identification of humpback whale non-song vocalizations

**Figure 1:**
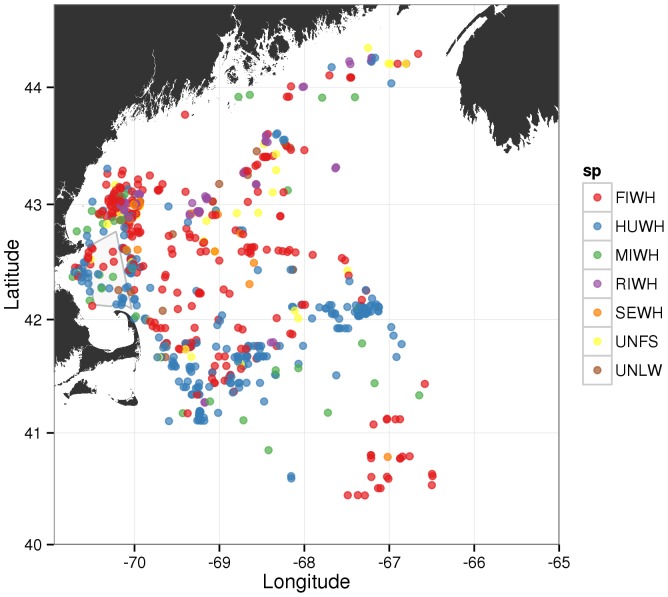
Sightings of baleen whales in the Gulf of Maine during right whale aerial surveys conducted by the Northeast Fisheries Science Center (NEFSC) during September 15 to October 17, for years: 1998, 2002–2008 and 2010. FIWH  =  fin whale, HUWH  =  humpback whale, MIWH  =  minke whale, RIWH  =  right whale, SEWH  =  sei whale, UNFS  =  unidentified fin or sei whale, UNLW  =  unidenitified large whale.

While humpback whale song has been well studied in the Gulf of Maine [8]–[11] and is easily distinguishable from other marine mammal sounds, other social or feeding vocalizations have been studied far less intensively (but see [12], [13]). The Gulf of Maine is a summer feeding ground for several baleen whale species, including sei, fin, right and minke whales [14]–[16]. Vocal repertoires for all of these species in the western North Atlantic have not been thoroughly described yet. Given the range overlap of these species in the Gulf of Maine (Figure 1) and the possibility of false species assignment, it is thus important to provide sufficient background, when describing new call types and interpreting species' behavior using solely passive acoustic data.

In marine mammal bioacoustics, the standard methods to describe new types of vocalizations are to either (1) verify species ID with visual sightings of the vocalizing animal [17], [18], (2) use recordings from acoustic tags [12], [19] or (3) compare recorded vocalizations with stereotyped vocalizations, recorded in the same geographic region, that have been matched to the species in question previously [16], [20]. Gong et al. [1] have not used any of these standard methods to verify that North Atlantic humpback whales produce “meows”, “bow-shaped calls” or “feeding cries”. Although these calls have not been described for any other species, it is conceivable that minke, sei, fin or right whales in the Gulf of Maine may produce similar tonal call types. Gong et al. [1] claimed that non-song calls were identified to species based on similar methods reported in Dunlop et al. [18]. However, contrary to Gong et al. [1], who based their analysis entirely on passive acoustic data, the study by Dunlop et al. [18], characterizing humpback whale social sounds from eastern Australia, used visual and acoustic localization techniques to verify not only species but to identify which group of humpback whales was vocalizing.

Further, Gong et al. state: “Since all non-song calls or non-song call sequences we detected consistently originated or ended at the same spatial position as song calls, to within our reported position error in Section 3.3, and occurred immediately after or before these co-located song calls, alternating with song calls, it is most likely that the same species and group of whales produced the song and non-song calls we report.” [1]. There are several problems with this justification. Firstly, Gong et al. [1] do not present any data to support this statement, making it impossible to assess these data. Yet, humpback whale songs typically follow a very rigid and stereotypical temporal pattern and are rarely interrupted, preceded or followed directly by non-song calls [21], [22]. Secondly, a position error of equal to or more than 1–2 km, as reported by Gong et al. [1], would easily allow for other nearby individuals of other species to produce these calls. In addition, Gong et al. state: “There were numerous sightings of humpback whales at Georges Bank during the 2006 Gulf of Maine experiment.” [1]. However, this statement is also very vague and similarly does not exclude the presence of other species in the same general area. Here again, the authors do not present any description of the methods used to confirm their visual surveys, or present data of their visual sightings. Again, it is therefore impossible to assess the extent to which their data support the validity of their conclusion. In contrast, Figure 1 illustrates multi-year aerial surveys conducted by the Northeast Fisheries Science Center during the months of September and October, which typically sight many humpback whales on Georges Bank, but other species, in particular fin and minke whales are also frequently sighted in the same general area. For details of these surveys' design and execution, see [23]. Thus, the claims, that North Atlantic humpback whales unequivocally produce the newly described non-song calls presented in Gong et al. [1] are questionable. These claims require substantially more supporting evidence to rule out that those sounds were produced by other species.

### 2. Suggestion of feeding related vocalizations and echolocation

Gong et al. suggest, that “*repetitive non-song calls*” which they ascribe to humpback whales (but see above), are “*consistent with […] prey echolocation during feeding activities*” [1]. However, the literature that Gong et al. [1] cite here are for odontocetes, which produce signals of very different spectral and temporal characteristics. The authors mention the recent suggestion that humpback whale “*Megapclicks*” may be used for rough acoustic sensing of the environment [12]. However, in their paper, Stimpert et al. specifically point out, that the acoustic properties of “*Megapclicks*” are “*unlikely properties for signals with an echolocation function*.”[12]. There is to our knowledge no other strong evidence for echolocation in humpback whales or any other baleen whale species. The suggestion of such a new concept, even if, as Gong et al. [1] argue, theoretical possible, should be presented much more carefully and in context of the relevant literature on baleen whale hearing and vocalization behavior, much of which has been omitted by Gong et al. [1].

Finally, the authors compare recorded “*feeding cries*” with data from Alaska [24] and state that these calls are evidence for cooperative feeding behavior. While the characteristics of these calls are similar to Alaskan feeding cries, these calls have not been described for humpback whales in the North Atlantic yet. More data are needed to back up the claim that these vocalizations are actually produced by humpback whales in the North Atlantic and in the suggested context (see above). In the absence of it, the suggestion of humpback whale cooperative feeding behavior on Georges Bank based on passive acoustics alone is not supported by the presented data.

In conclusion, given the substantial uncertainties with respect to sound identification and behavioral context, Gong et al.'s [1] data on synchronous herring shoaling and humpback whale vocalizations is a potentially interesting finding. However, the methods, results and conclusions remain unconvincing until further validation work is carried out.

## Critique of Data and Analysis Presented in Risch et al. 2012 [2]

### 1. Humpback whale song localization and suggested lack of vocalizations originating on Stellwagen Bank

**Figure 2:**
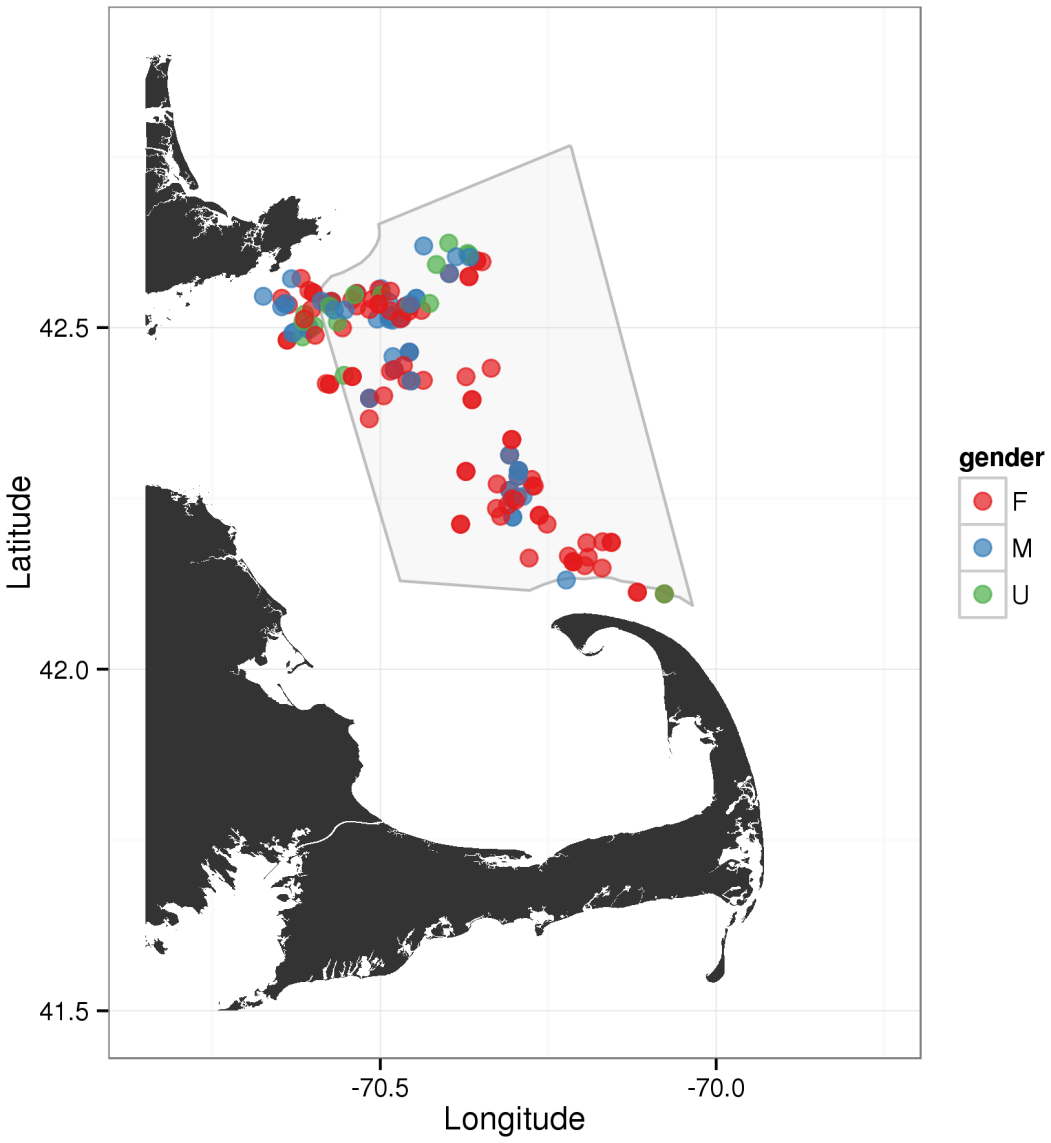
Sightings of individually identified humpback whales by sex (one location/day; dependent calves excluded) in the Stellwagen Bank area from September 22 to October 6, 2006. Data courtesy Jooke Robbins (CCS), collected by Provincetown Center for Coastal Studies (CCS) and the Whale Center of New England (WCNE).

In paragraph 3.5 of their Methods section, Gong et al. state that “The fact that we localized the sources of many whale calls on Georges Bank but found negligibly small vocalization rates originating from Stellwagen Bank in the “before” or “during” periods, then emphasizes the fact that vocalization rates originating from Stellwagen Bank were negligibly small in these periods.” [1]. This statement contradicts the following statements made in the Abstract and Introduction of the paper, respectively: “[…] no vocalizing whales were found at Stellwagen Bank […]”; “[…] our data shows no humpback whale vocal activity originating from Stellwagen Bank […]” [1], indicating that zero humpback whale sounds were detected on Stellwagen Bank. With this contradiction and without actual numbers of localized calls and their locations (these data are impossible to infer from the large-scale call density maps presented in Figures 1-3,13 in Gong et al. and are not listed elsewhere in the paper [1]), it is impossible to evaluate differences in vocalization rates between this and our earlier study on humpback whale song recorded on Stellwagen Bank [2], as Gong et al. [1] have done.

Between September 22 and October 6, 2006, researchers from the Provincetown Center for Coastal Studies (CCS) and the Whale Center of New England (WCNE) identified 68 individual humpback whales in the Stellwagen Bank area, with a total of 19 males, 14 of which were mature at the time of the sighting (Figure 2, Table 1, J. Robbins pers. communication). It is highly unlikely, that, during this time period, the whales residing in SBNMS would have not been vocalizing, as Gong et al. [1] imply. This seems especially true if one takes song and non-song vocalizations into account as Gong et al. have apparently done [1]. The same is likely true for whales in areas in the northern Gulf of Maine that are presumably also in the OAWRS detection range area (Figures 1-3,13 in [1]). While a concentration of animals and therefore vocalizations might have been found on Georges Bank, some vocalizations can be expected to have been produced in other areas of the Gulf of Maine. Gong et al.'s finding of almost no to no vocalizations in these other areas [1] suggests that the authors may be considerably overestimating their own detection and localization range. This further suggests that their data cannot support the claim that no humpback whale vocalizations originated on Stellwagen Bank before and during the 2006 OAWRS experiment.

**Table 1:** Sightings of individually identified humpback whales by sex (one location/day; dependent calves excluded) in the Stellwagen Bank area from September 22 to October 6, 2006.

Whale ID	Date	Time	Latitude	Longitude	Sex
14	9/22/2006	11∶35	42.16267	−70.2785	F
14	9/25/2006	13∶57	42.1655	−70.22	F
17	9/22/2006	18∶22	42.26167	−70.3075	F
34	9/26/2006	17∶35	42.18583	−70.1565	F
34	9/27/2006	14∶28	42.23517	−70.3272	F
34	10/04/2006	14∶49	42.247	−70.299	F
58	9/22/2006	11∶01	42.3665	70.5164	F
58	9/23/2006	11∶46	42.41697	70.57567	F
58	9/26/2006	14∶01	42.11267	−70.1173	F
60	9/25/2006	14∶12	42.1865	−70.169	F
60	9/26/2006	10∶51	42.148	−70.1698	F
60	9/27/2006	11∶13	42.27767	−70.2747	F
60	9/28/2006	11∶07	42.271	−70.326	F
60	10/03/2006	14∶08	42.185	−70.1925	F
78	9/22/2006	10∶24	42.15717	−70.213	F
78	9/24/2006	11∶20	42.11017	−70.0772	F
108	9/22/2006	17∶59	42.25177	−70.2954	F
128	9/28/2006	11∶30	42.39444	70.36345	F
139	9/30/2006	16∶29	42.44051	70.33506	F
143	10/02/2006	14∶11	42.1305	−70.2232	M
178	9/30/2006	10∶44	42.53137	70.46198	M
200	9/25/2006	14∶28	42.1855	−70.1557	F
200	9/26/2006	17∶18	42.164	−70.1905	F
206	9/23/2006	14∶20	42.4284	70.54181	F
259	9/23/2006	10∶50	42.464	70.45671	F
296	9/27/2006	10∶51	42.268	−70.2737	F
297	9/23/2006	10∶29	42.1515	−70.1957	F
297	9/27/2006	14∶05	42.24017	−70.3103	F
297	9/30/2006	11∶11	42.33617	−70.3043	F
304	9/22/2006	10∶24	42.15717	−70.213	F
304	9/25/2006	15∶01	42.44442	70.46567	F
363	10/02/2006	12∶44	42.57912	70.39718	F
446	9/23/2006	15∶42	42.43612	70.48503	F
481	9/22/2006	12∶25	42.48172	70.63874	F
481	9/30/2006	15∶50	42.42198	70.45405	F
481	10/02/2006	15∶19	42.42804	70.37249	F
481	10/03/2006	11∶24	42.28944	70.37253	F
528	9/22/2006	14∶27	42.22267	−70.3028	M
528	9/23/2006	10∶50	42.464	70.45671	M
533	9/23/2006	11∶46	42.41697	70.57567	F
533	9/30/2006	13∶59	42.22517	−70.2633	F
594	9/28/2006	13∶26	42.51433	70.46752	F
600	9/22/2006	10∶55	42.21233	−70.2518	F
600	9/29/2006	14∶24	42.28217	−70.295	F
600	9/30/2006	10∶53	42.3135	−70.3075	F
600	10/04/2006	14∶15	42.29067	−70.2943	F
636	9/24/2006	15∶13	42.5317	70.53478	F
1079	9/22/2006	15∶06	42.22383	−70.321	F
1079	9/23/2006	14∶20	42.4284	70.54181	F
1080	9/28/2006	11∶30	42.39444	70.36345	F
1080	9/30/2006	11∶11	42.33617	−70.3043	F
1109	9/22/2006	14∶27	42.22267	−70.3028	M
1109	9/28/2006	10∶43	42.2495	−70.3035	M
1109	9/29/2006	14∶24	42.28217	−70.295	M
1109	9/30/2006	10∶53	42.3135	−70.3075	M
1109	10/02/2006	11∶43	42.43846	70.4794	M
1109	10/04/2006	14∶15	42.29067	−70.2943	M
1129	9/24/2006	11∶45	42.45742	70.48117	M
1129	9/26/2006	13∶18	42.53812	70.57233	M
1158	9/25/2006	12∶06	42.52678	70.60218	F
1166	9/26/2006	14∶01	42.11267	−70.1173	F
1166	9/29/2006	10∶46	42.26817	−70.2708	F
1186	9/30/2006	15∶50	42.42198	70.45405	M
1186	10/02/2006	12∶44	42.57912	70.39718	M
1186	10/04/2006	14∶15	42.29067	−70.2943	M
1203	9/30/2006	12∶31	42.52521	70.43866	F
1205	9/22/2006	12∶25	42.48172	70.63874	F
1205	9/30/2006	13∶59	42.22517	−70.2633	F
1211	9/22/2006	18∶22	42.26167	−70.3075	M
1211	9/28/2006	13∶38	42.51292	70.47018	M
1220	9/30/2006	14∶40	42.21217	−70.3805	F
1241	10/02/2006	11∶43	42.43846	70.4794	F
1241	10/03/2006	11∶24	42.28944	70.37253	F
1247	9/22/2006	10∶24	42.15717	−70.213	F
1309	9/23/2006	11∶39	42.49956	70.5563	F
1309	9/26/2006	11∶45	42.51253	70.61308	F
1309	9/27/2006	11∶10	42.40011	70.49497	F
1309	9/28/2006	14∶15	42.52462	70.48516	F
1309	9/30/2006	14∶40	42.21217	−70.3805	F
1328	9/23/2006	10∶50	42.464	70.45671	M
1339	9/22/2006	11∶34	42.39643	70.51601	F
1341	9/26/2006	11∶52	42.5404	70.54244	F
1342	9/22/2006	11∶34	42.39643	70.51601	F
1342	9/23/2006	13∶55	42.41797	70.58052	F
1342	9/25/2006	15∶23	42.42281	70.43594	F
1343	9/26/2006	14∶01	42.53876	70.58771	M
1343	9/27/2006	12∶59	42.51242	70.48706	M
1345	9/28/2006	12∶55	42.54782	70.4978	F
1345	9/30/2006	10∶44	42.53137	70.46198	F
1346	10/04/2006	14∶39	42.60298	70.36697	M
1368	9/22/2006	11∶34	42.39643	70.51601	M
1368	9/30/2006	16∶31	42.53491	70.45544	M
1423	9/22/2006	13∶03	42.51977	70.61253	U
1423	9/23/2006	15∶03	42.48629	70.61575	U
1423	9/25/2006	13∶11	42.50228	70.59851	U
1423	9/26/2006	13∶11	42.53812	70.57233	U
1452	9/22/2006	14∶02	42.57145	70.63231	M
1452	9/28/2006	11∶36	42.52557	70.56867	M
1461	9/22/2006	11∶42	42.5429	70.64671	F
1461	9/28/2006	13∶38	42.51292	70.47018	F
1461	9/30/2006	11∶41	42.52373	70.45778	F
1461	10/04/2006	12∶05	42.59633	70.34866	F
1462	9/22/2006	11∶54	42.53497	70.64028	M
1462	9/23/2006	14∶04	42.49381	70.62718	M
1462	9/24/2006	14∶02	42.54581	70.67416	M
1462	9/27/2006	11∶57	42.52568	70.55273	M
1462	10/03/2006	12∶31	42.61947	70.43549	M
1471	9/23/2006	16∶20	42.42299	70.46044	F
1472	9/25/2006	12∶47	42.50179	70.60646	M
1472	9/28/2006	15∶08	42.253	−70.2875	M
1487	9/22/2006	14∶34	42.55446	70.60639	F
1487	9/24/2006	15∶21	42.5363	70.57889	F
1487	9/25/2006	13∶13	42.48865	70.59696	F
1487	9/26/2006	13∶18	42.53812	70.57233	F
1487	9/27/2006	11∶14	42.51778	70.48332	F
1487	9/28/2006	15∶34	42.53421	70.50288	F
1487	9/30/2006	10∶16	42.55325	70.48489	F
1487	10/04/2006	12∶27	42.59778	70.35644	F
1569	9/28/2006	10∶43	42.2495	−70.3035	F
1577	9/26/2006	11∶48	42.54999	70.53497	M
1577	9/27/2006	12∶29	42.5163	70.48749	M
1577	9/30/2006	14∶52	42.54277	70.44643	M
1588	9/23/2006	14∶15	42.43006	70.55384	U
1588	10/04/2006	11∶11	42.59229	70.41653	U
1648	9/22/2006	11∶29	42.53343	70.63686	F
1648	9/24/2006	14∶59	42.53811	70.57375	F
1648	9/25/2006	11∶47	42.55096	70.59983	F
1648	9/26/2006	11∶48	42.54999	70.53497	F
1648	9/27/2006	11∶14	42.51778	70.48332	F
1648	9/28/2006	15∶34	42.53421	70.50288	F
1648	9/30/2006	14∶54	42.5314	70.45548	F
1648	10/03/2006	11∶00	42.57449	70.36803	F
1648	10/04/2006	12∶27	42.59778	70.35644	F
1673	9/27/2006	12∶43	42.51076	70.48193	M
1673	9/28/2006	13∶12	42.53745	70.48735	M
1776	9/22/2006	12∶08	42.53044	70.64691	M
1776	9/30/2006	14∶52	42.54277	70.44643	M
1783	9/28/2006	12∶33	42.55699	70.49895	M
1799	9/25/2006	14∶00	42.50926	70.61517	U
1799	9/26/2006	14∶23	42.53139	70.57544	U
1799	9/27/2006	11∶14	42.51778	70.48332	U
1799	9/30/2006	15∶58	42.53529	70.42661	U
1799	10/03/2006	14∶59	42.60854	70.37003	U
1856	9/25/2006	16∶27	42.50815	70.56398	U
1856	9/30/2006	09∶41	42.54821	70.53838	U
1886	9/22/2006	11∶33	42.53478	70.64262	M
1886	9/23/2006	15∶31	42.4972	70.60645	M
1886	9/24/2006	15∶28	42.49179	70.6306	M
1886	9/27/2006	14∶17	42.51242	70.50469	M
1886	10/04/2006	15∶05	42.60363	70.38695	M
1912	9/22/2006	13∶40	42.57193	70.61803	F
1912	9/24/2006	14∶59	42.53811	70.57375	F
1912	9/25/2006	11∶47	42.55096	70.59983	F
1912	9/26/2006	12∶54	42.54142	70.5116	F
1912	9/27/2006	14∶37	42.52653	70.51601	F
1912	9/28/2006	12∶38	42.55573	70.50218	F
1912	9/30/2006	11∶59	42.53275	70.4566	F
1912	10/03/2006	11∶00	42.57449	70.36803	F
1939	10/03/2006	16∶00	42.62359	70.39815	U
1939	10/04/2006	12∶48	42.60649	70.36782	U
1944	9/28/2006	12∶55	42.54782	70.4978	U
1944	9/30/2006	11∶59	42.53275	70.4566	U
2010	9/24/2006	11∶20	42.11017	−70.0772	U

Data courtesy Jooke Robbins (CCS), collected by Provincetown Center for Coastal Studies (CCS) and the Whale Center of New England (WCNE).

It is possible that some of the reduction of humpback whale song reported in Risch et al. [2] might have been related to changes in environmental conditions. However, for the reasons outlined above and as we did not observe large changes in ambient noise levels over the entire time period [2], we are confident in our inference that the changes in humpback whale song we reported were due to changes in occurrence of song originating on Stellwagen Bank. These changes cannot be explained by changes in wind speed and the occurrence of song 50–150 km from our study site.

### 2. Statistical test and interpretation of results

Variability in humpback whale song occurrence is commonly observed and has been shown for this area by Vu et al. [10]. Singing humpback whales arrive in SBNMS in the spring and generally stop singing during summer when they are engaged in foraging. During this time humpback whales produce mainly lesser-known social and feeding-associated sounds, while humpback whale song production is very variable [10]. Humpback whales start singing again in the fall season just before their southerly migration. During this time the increase in recorded humpback whale song is substantial. This is demonstrated in the 2008 and 2009 data we collected (Figure 3 [2]). Given this seasonal variability in mean humpback whale song occurrence, comparing 11-day periods across the whole year [1] and comparing the outcome to our results, which are focused on (a) the fall singing period, and (b) using the period during which the OAWRS signal was being broadcast [2], is therefore misleading, as it masks expected trends in song occurrence.

### 3. Violation of temporal causality?

Gong et al. [1] state that the fact that we report reduced numbers of minutes with humpback whale song before the start of the full 2006 OAWRS transmission shows a violation of temporal causality. However, this reduction of song two days prior to the experiment could have just been due to random fluctuations in daily whale numbers in the detection range of our acoustic sensor. Varying minutes with humpback whale song from one day to the next are in the realm of natural fluctuations and may be related to individual whale movement or, as Gong et al. [1] point out, can be related to environmental conditions influencing detection range. Thus, in order to smooth out expected daily fluctuations in song occurrence, our analysis looked at an 11-day time series before, during and after the OAWRS experiment and compared the 2006 data to two other years to compare the overall temporal trend of increasing song presence at the start of the fall migration. To reiterate, we treated the OAWRS signal as a treatment effect in our analysis. Our main result was to show that, compared to the two years when the OAWRS signal was not broadcast, humpback whale song in 2006 did not increase consistently during this 33-day period and that the reduced presence of humpback whale song from September 26 to October 6, 2006 coincided with the presence of the OAWRS signal recorded in our data.

## Discussion of the Effects of Anthropogenic Noise

### 1. Effects of OAWRS transmissions on humpback whale song occurrence and variability of behavioral responses to anthropogenic noise

As pointed out earlier, several humpback whales were observed in the SBNMS area before and during the 2006 OAWRS experiment (Figure 2, Table 1). However, Gong et al. [1] report no vocalizations from this area over this entire time period. Given the number of animals and typical calling rates for humpback whales, these results are highly unlikely and indicate that Gong et al.'s claim: *“[…] transmissions had no effect on humpback whale song over the entire passive 400 km diameter survey area of the Gulf of Maine including Stellwagen Bank.*” (Figure 9 in [1]) is not supported by their data.

In addition, while Gong et al. might have measured constant song occurrence on Georges Bank close to the OAWRS source array [1], these results do not contradict our findings from recordings made on Stellwagen Bank. Whales' behavioral responses to noise are complex and may change with behavioral context, prey availability, distance from source, RL, signal structure and novelty, as well as differences elicited by individual whales [5], [6]. In Risch et al. [2] we clearly state that possible contextual reasons for the observed behavioral changes include the relatively short duration of the OAWRS experiment, as well as the novelty of the OAWRS pulses in combination with low RLs, 200 km from the source.

Although not measured by Gong et al. [1], it is likely that humpback whales that were feeding on Georges Bank, in close proximity to the OAWRS source array, were exposed to much higher RLs from OAWRS, than singing humpback whales on Stellwagen Bank [7]. Different behavioral responses to the same noise source are thus expected, especially given that there were likely differences in general behaviors between the two sites. While both sites are feeding sites for humpback whales at this time of year, differences in prey abundance, especially the presence of large herring shoals on Georges Bank [1] might have contributed to the difference in response to the OAWRS signals.

In addition, responses to the OAWRS signal could have been different from song production rates alone. For example, Miller et al. [25] showed that humpback whales increased song duration during exposure to LFA sonar. A similar response in the presence of OAWRS source signals could partly explain the observed differences between our study and Gong et al. [1]. With our limited dataset, that did not allow localization of individual whales, we were not able to assess behavioral parameters other than song occurrence. In contrast, while Gong et al. [1] had the data for it (i.e. they localized individual humpback whales); they provide no further behavioral analyses to assess whether humpback whales' responses to the 2006 OAWRS experiment varied over time and space.

### 2. Impact of anthropogenic noise on marine mammals

In their Discussion Gong et al. [1] point out the importance of assessing all sources of noise, including background noise, when assessing the impact of a particular noise source on the marine environment. We strongly agree with this view. However, we recorded the OAWRS signal 200 km from its source serendipitously. Therefore our aim was not a full assessment of the impact of the OAWRS signal source on marine mammal behavior, for which, contrary to Gong et al. [1], we also did not have the data. In contrast, to our knowledge, such an assessment of the OAWRS source signal has not been conducted by the authors of Gong et al. [1], two of which also hold patent US 2006/0280030 with respect to *“Continuous, continental-shelf-scale monitoring of fish populations and behavior”* using OAWRS.

In our paper we discussed that the observed behavioral response of singing humpback whales occurred towards very low RLs of OAWRS signals recorded on Stellwagen Bank, roughly 200 km from the signal source [2]. Due to these low levels, one of the main points of the paper was to highlight the importance of behavioral context, ambient noise, as well as novelty of sounds when assessing anthropogenic noise impacts on marine mammals. While noise impacts have traditionally been assessed based on dose-response models, a behavioral response to signals with very low signal excess, as presented by Risch et al. [2], highlights the need for new approaches to assessing noise exposure risks.

High levels of unexplained variability within and between individual animals' responses to noise have been pointed out in several recent studies on dose-response relationships [26], [27]. And several authors and agencies have started to discuss the notion that absolute noise levels alone are insufficient to accurately assess the variability in responses to anthropogenic noise, that go far beyond the risks of acute injury of marine mammals [5], [6]. Recent data on the impact of military sonar on blue and Cuvier's beaked whales support this idea and show that both baleen and toothed whales may respond to lower than currently regulated RLs of military sonar signals [28], [29].

To reiterate, a differential response of humpback whales, which were closer to the sound source and exposed to higher RLs (i.e. whales feeding on Georges Bank) as compared to those that were further away (i.e. whales feeding in SBNMS), would be an interesting finding, rather than a contradiction. This result is noteworthy but was not explored by Gong et al. [1].
